# *Ex Vivo* Characterization of Effects of Renal Replacement Therapy Modalities and Settings on Pharmacokinetics of Meropenem and Vaborbactam

**DOI:** 10.1128/AAC.01306-18

**Published:** 2018-09-24

**Authors:** Fekade B. Sime, Saurabh Pandey, Nermin Karamujic, Suzanne Parker, Elizabeth Alexander, Jeffery Loutit, Stephanie Durso, David Griffith, Jeffrey Lipman, Steven C. Wallis, Jason A. Roberts

**Affiliations:** a School of Pharmacy, Centre for Translational Anti-infective Pharmacodynamics, The University of Queensland, Brisbane, Australia; b University of Queensland Centre for Clinical Research, Faculty of Medicine, The University of Queensland, Brisbane, Australia; c The Medicines Company, San Diego, California, USA; d Department of Intensive Care Medicine, Royal Brisbane and Women's Hospital, Brisbane, Australia; e Pharmacy Department, Royal Brisbane and Women's Hospital, Brisbane, Australia

**Keywords:** renal replacement therapy, extracorporeal clearance, adsorption, meropenem, vaborbactam

## Abstract

The combination product meropenem-vaborbactam, with activity against KPC-producing carbapenem-resistant Enterobacteriaceae, is likely to be used during renal replacement therapy. The aim of this work was to describe the extracorporeal removal (adsorption and clearance) of meropenem-vaborbactam during continuous venovenous hemofiltration (CVVH). An *ex vivo* model was used to examine the effects of a matrix of operational settings. Vaborbactam did not adsorb to AN69 (acrylonitrile and sodium methallylsulfonate copolymer) ST100 (surface area, 1 m^2^) hemofilter; the mean (±standard deviation [SD]) meropenem adsorption was 9% (±1%). The sieving coefficients (mean ± SD) with AN69 ST100 and ST150 (surface area, 1.5 m^2^) filters ranged from 0.97 ± 0.16 to 1.14 ± 0.12 and from 1.13 ± 0.01 to 1.53 ± 0.28, respectively, for meropenem and from 0.64 ± 0.39 to 0.90 ± 0.14 and 0.78 ± 0.18 to 1.04 ± 0.28, respectively, for vaborbactam. At identical settings, vaborbactam sieving coefficients were 25% to 30% lower than for meropenem. Points of dilution, blood flow rates, or effluent flow rates did not affect sieving coefficients for either drug. However, doubling the effluent flow rate resulted in >50 to 100% increases in filter clearance for both drugs. Postfilter dilution resulted in 40 to 80% increases in filter clearance at a high effluent flow rate (4,000 ml/h), compared with ∼15% increases at a low effluent flow rate (1,000 ml/h) for both drugs. For all combinations of setting and filters tested, vaborbactam clearance was lower than that of meropenem by ∼20 to 40%. Overall, meropenem-vaborbactam is efficiently cleared in CVVH mode.

## Abstract (precis)

The combination product meropenem-vaborbactam, with activity against KPC-producing carbapenem-resistant Enterobacteriaceae, is likely to be used during renal replacement therapy. The aim of this work was to describe the extracorporeal removal (adsorption and clearance) of meropenem-vaborbactam during continuous venovenous hemofiltration (CVVH).

## INTRODUCTION

Carbapenem-resistant Gram-negative bacteria are at the top of World Health Organization's (WHO) priority list for research and development of novel antibiotics (1). One promising novel product recently developed is the combination product meropenem with vaborbactam. Vaborbactam is a serine beta-lactamase inhibitor that prevents hydrolysis of carbapenem antibiotics by bacterial enzymes (carbapenemases) (2), the most common mechanism for the emergence of carbapenem-resistant Enterobacteriaceae (CRE) (3). In August 2017, the U.S. Food and Drug Administration (FDA) approved the fixed-dose meropenem-vaborbactam combination product Vabomere for the management of complicated urinary tract infections (UTI) (4), following a favorable outcome in a noninferiority trial (5). This meropenem-vaborbactam combination product has gained significant interest due to its activity against KPC-producing Enterobacteriaceae, which are spreading worldwide and for which existing antibiotics offer limited efficacy (6). Compared to current best therapy for KPC-producing CRE infections, the meropenem-vaborbactam combination has shown promising advantages in terms of higher clinical cure rates (7).

Patients with KPC-producing CRE infections may develop severe renal impairment that necessitates the use of continuous renal replacement therapy (CRRT) (8). Thus, the use of meropenem-vaborbactam in patients undergoing CRRT is likely. To date, there is accumulating evidence for the need to adjust dosing regimens of meropenem and other antibiotics according to the CRRT operational settings and the degree of residual renal function (9, 10). Empirical-dosing regimens are unlikely to meet optimal dosing targets in a significant proportion of patients receiving CRRT due to the altered and variable extracorporeal clearances (10, 11). Unfortunately, the impact of CRRT on drug clearance is difficult to predict due to the various contributing factors, including filter type and surface area, blood flow rate, effluent flow rate, replacement fluid settings (or point of dilution), mechanisms of filtration (or CRRT modalities) (12) and also sequestration of drug molecules within the CRRT circuit system (13). It is therefore important to test the effect of individual factors in a controlled experiment whereby a single study-variable/operating parameter is altered at a time to describe its pharmacokinetic effects on dosing requirements. In this regard, few studies exist for the impact of CRRT on the clearance of meropenem (14–19), and no studies exist for the new agent vaborbactam.

The aims of this work were (i) to estimate the extent of adsorption of meropenem and vaborbactam within a clinically used continuous venovenous hemofiltration (CVVH) circuit system and (ii) to describe the effect of point of dilution and a range of common CVVH settings on the extracorporeal removal of meropenem and vaborbactam.

## RESULTS

Given the difficulty of conducting a clinical study in which the various operational settings of CVVH are altered in a controlled manner so as to describe direct effects on adsorption and/or clearance of drugs, we used an *ex vivo* model of CVVH with a clinically used CRRT machine and human blood-crystalloid mixture to closely simulate the clinical condition. The *ex vivo* model was set up with the commonly used AN69 (a copolymer of acrylonitrile and sodium methallylsulfonate) hemofilters, ST100 (surface area, 1 m^2^) or ST150 (surface area, 1.5 m^2^).

**FIG 1:**
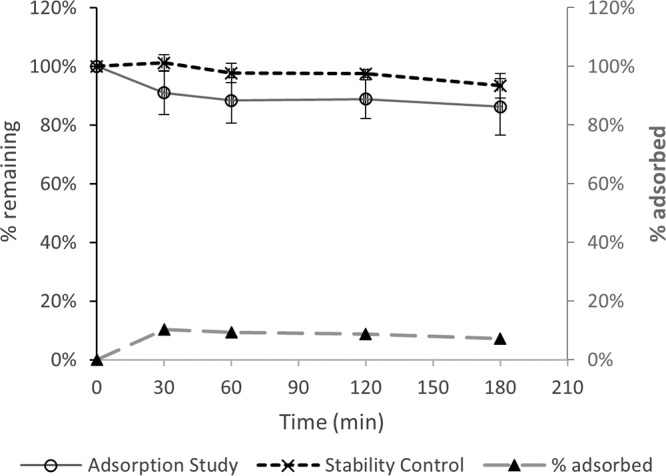
Adsorption of meropenem onto an AN69 filter (50 mg/liter at 100- and 200-ml/min blood flow rates); comparison with stability over 3 h at 37°C.

**FIG 2:**
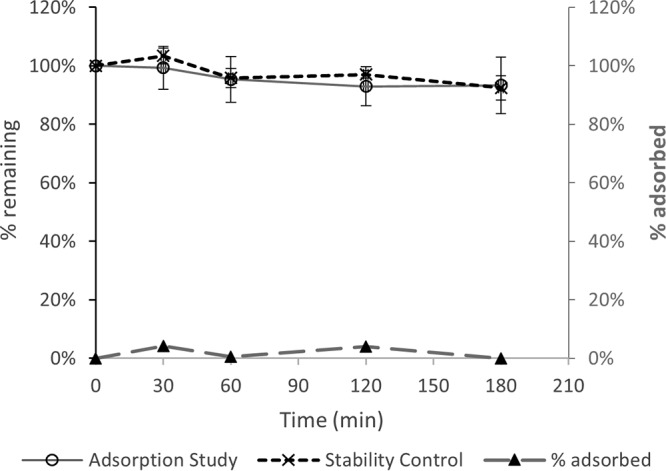
Adsorption of 50 mg/liter of vaborbactam onto an AN69 filter (50 mg/liter at 100- and 200-ml/min blood flow rates); comparison with stability over 3 h at 37°C.

Initial “plasma” concentrations (mean ± standard deviation [SD]) measured from blood-crystalloid mixtures prepared at 50 mg/liter were 53.4 ± 3.5 mg/liter and 60.2 ± 7.3 mg/liter for meropenem and vaborbactam, respectively. These plasma concentrations, which do not affect result analysis, are expected given that the hematocrit of the blood-crystalloid mixture was approximately 20% and the possibility of red blood cell partitioning. For a blood-crystalloid mixture spiked to 50 mg/liter, circulating around a closed ST100 circuit over 3 h, the meropenem concentration decreased relative to that in the stability control experiment (Fig. 1). The percentages of meropenem remaining at 3 h were similar at different initial concentrations (86% ± 10% at 50 mg/liter and 88% ± 12% at 5 mg/liter). For vaborbactam, however, the percent drug remaining during the adsorption experiment was very close to that of the stability control (Fig. 2). The different blood flow rates (200 ml/min versus 100 ml/min) and initial concentrations (50 mg/liter versus 5 mg/liter) resulted in comparable percentages of drug remaining after 3 h of circulation within the RRT circuit (Table 1). Figures 1 and 2 also compare the mean percentages of drug remaining in the mixing chamber during the adsorption study with the estimated percent adsorption. For meropenem, the percent adsorption ranged from 7 to 10% over the 3 h of circulation and averaged 9%. For vaborbactam, on the other hand, the estimated adsorption averaged 2%, which suggests no appreciable adsorption when considering experimental noise.

**TABLE 1:** Comparison of percentages of drug remaining in the mixing chamber during the adsorption experiment by initial drug concentration or blood flow rate

Initial concn (mg/liter)	Blood flow rate (ml/min)	% drug remaining at 3 h (mean ± SD)	
		Meropenem	Vaborbactam
50	200	89 ± 13	91 ± 5
	100	85 ± 9	95 ± 7
5	200	83 ± 10	96 ± 6
	100	102 (*n* = 1)^*a*^	98 (*n* = 1)^*a*^

a For all results, the experiments was repeated three times except for the 5-mg/liter initial concentration with blood flow rate of 100 ml/min, for which the experiment was performed only once.

Tables 2 and 3 compare the effects of point of dilution on meropenem and vaborbactam sieving coefficients for the ST100 and ST150 filters at different blood flow rates and effluent flow rates. Sieving coefficients were not affected by points of dilution, blood flow rates, or effluent flow rates. At identical settings, vaborbactam sieving coefficients were 25% to 30% lower than that of meropenem.

**TABLE 2:** Effect of point of dilution on meropenem-vaborbactam sieving coefficients of the AN69 ST100 filter at different blood flow rates and effluent flow rates

Blood flow rate (ml/min)	Effluent flow rate (liters/h)	Sieving coefficient by drug and point of dilution (mean ± SD)			
		Meropenem		Vaborbactam	
		Prefilter	Postfilter	Prefilter	Postfilter
200	4	1.05 ± 0.09	1.08 ± 0.17	0.78 ± 0.10	0.83 ± 0.16
	2	1.14 ± 0.12	1.07 ± 0.02	0.88 ± 0.15	0.85 ± 0.12
	1	1.10 ± 0.06	1.07 ± 0.09	0.90 ± 0.14	0.86 ± 0.15
100	4	1.06 ± 0.16	0.97 ± 0.16	0.64 ± 0.39	0.70 ± 0.16
	2	1.01 ± 0.13	1.08 ± 0.14	0.79 ± 0.16	0.78 ± 0.12
	1	1.02 ± 0.12	1.02 ± 0.08	0.80 ± 0.14	0.81 ± 0.10

**TABLE 3:** Effect of point of dilution on meropenem-vaborbactam sieving coefficients of the AN69 ST150 filter at a blood flow rate of 200 ml/min and different effluent flow rates

Effluent flow rate (liters/h)	Sieving coefficient by drug and point of dilution (mean ± SD)			
	Meropenem		Vaborbactam	
	Prefilter	Postfilter	Prefilter	Postfilter
4	1.28 ± 0.14	1.18 ± 0.09	0.80 ± 0.16	0.78 ± 0.18
2	1.16 ± 0.09	1.53 ± 0.28	0.85 ± 0.15	1.04 ± 0.28
1	1.13 ± 0.01	1.18 ± 0.06	0.82 ± 0.24	0.80 ± 0.15

**FIG 3:**
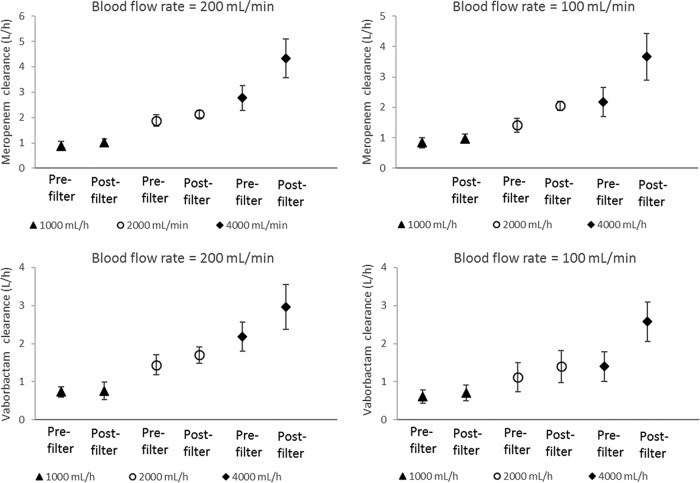
Effect of point of dilution on meropenem and vaborbactam filter clearance (with ST100 filter) at different effluent flow rates and blood flow rates. The different markers indicate effluent flow rates.

**FIG 4:**
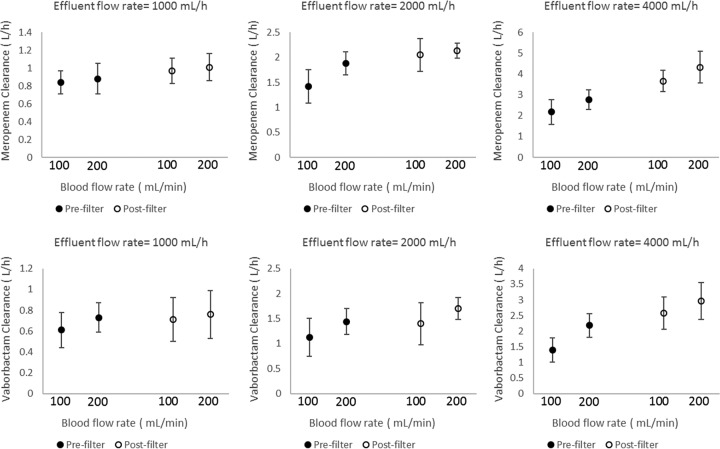
Effect of blood flow rate (100 versus 200 ml/min) on meropenem-vaborbactam filter clearance using an AN69 ST100 filter during prefilter and postfilter dilution and at different effluent flow rates. The different markers indicate point of dilution as either prefilter or postfilter.

The effect of point of dilution on meropenem and vaborbactam CVVH clearance (with ST100 filter) for different blood flow rates and effluent flow rates is illustrated in Fig. 3. Generally, postfilter fluid replacement resulted in higher extracorporeal clearance for both drugs, particularly at higher (4,000 ml/h) effluent flow rates (∼40 to 80% increases). However, at a lower (1,000 ml/h) effluent flow rate, clearances were comparable (within ∼15%) for pre- and postfilter dilution. Doubling the effluent flow rate resulted in more than 50 to 100% increases in CVVH clearance (Fig. 3). The effect of blood flow rate (100 versus 200 ml/min) on meropenem-vaborbactam clearance is illustrated in Fig. 4 (for the ST100 filter). At a given effluent flow rate, a blood flow rate of 200 ml/min resulted in numerically higher CVVH clearance than did blood flow rate of 100 ml/min for both pre- and postfilter dilution. The ST100 and ST150 resulted in comparable clearances for meropenem and vaborbactam (Table 4). However, vaborbactam clearance was consistently lower by ∼20 to 40% than that of meropenem for all combinations of setting and filters tested.

**TABLE 4:** Comparison of meropenem/vaborbactam clearances by AN69 ST100 and AN69 ST150 (blood flow rate, 200 ml/min)^*a*^

Effluent flow rate (liters/h)	Meropenem and vaborbactam clearance by point of dilution and type of filter (liters/h, mean ± SD)							
	Prefilter dilution				Postfilter dilution			
	Meropenem		Vaborbactam		Meropenem		Vaborbactam	
	ST150	ST100	ST150	ST100	ST150	ST100	ST150	ST100
4	2.35 ± 1.44	2.77 ± 0.48	1.38 ± 0.65	2.18 ± 0.38	3.48 ± 1.87	4.33 ± 0.77	2.02 ± 0.79	2.97 ± 0.59
2	1.84 ± 0.61	1.88 ± 0.23	1.38 ± 0.65	1.44 ± 0.26	2.39 ± 0.45	2.14 ± 0.15	1.34 ± 0.46	1.70 ± 0.22
1	0.99 ± 0.09	0.88 ± 0.17	0.69 ± 0.13	0.73 ± 0.14	0.94 ± 0.18	1.01 ± 0.15	0.54 ± 0.16	0.76 ± 0.23

a ST150, AN69 (a copolymer of acrylonitrile and sodium methallylsulfonate) hemofilter with a surface area of 1.5 m^2^; ST100, AN69 hemofilter with a surface area of 1 m^2^.

## DISCUSSION

Meropenem and vaborbactam were shown to exhibit similar *in vivo* pharmacokinetic properties with no interactions that affect each other's disposition, an important feature suitable for fixed-dose combination (20). This study examined their relative pharmacokinetics during CRRT *ex vivo*. Although a few clinical and *in vitro* studies have described the pharmacokinetics of meropenem during CRRT (14–19), this is the first study evaluating the extracorporeal removal of meropenem-vaborbactam by CVVH *ex vivo* under a range of operational settings for the clinically used AN69 ST100 and ST150 filters. The percent loss of the meropenem dose due to circuit sequestration in this study (mean, 9%) was the same as a previous estimate of 9% (14) for the Aquamax polysulfone filter and the Aquarius RRT circuit. However, the loss of less than 10% of the initial dose is unlikely to have any clinical consequence that requires dose adaptation for patients receiving CRRT. On the other hand, we did not observe any appreciable vaborbactam adsorption to the CVVH circuit/filter, similar to previous observations for another beta-lactamase inhibitor of different structure, tazobactam (21). Importantly, for both meropenem and vaborbactam, the extent of adsorption was not a zero-order kinetic process, with comparable percentages of drug loss (Table 1) for initial blood concentrations of 50 mg/liter and 5 mg/liter; this is very important because it means that at low concentrations, unintended excessive loss of the antibiotics is unlikely to occur during CVVH.

The mean meropenem sieving coefficients for the ST100 filter (0.97 to 1.14 [Table 2]) were similar to previously reported values from *ex vivo* studies, albeit with different filters and settings, for example, 1.06 to 1.17 for the Aquamax 1.2-m^2^ polysulfone filter (14), 1.05 for a polysulfone 0.75-m^2^ filter (16), and 0.88 for AN69 0.9-m^2^ and polysulfone 1.15-m^2^ filters (17). Similar reported values from clinical studies also exist for different filters and settings: 1.10 for Aquamax 1.2-m^2^ polysulfone filter (14), 0.93 for an AN69 0.9-m^2^ filter (19), and 1.09 for a polysulfone 0.43-m^2^ filter (18). For the ST150 filter, mean meropenem sieving coefficients were higher by up to ∼50% (1.13 to 1.53 [Table 3]), consistent with the 50% higher effective surface area of this filter (1.5 m^2^ versus 1 m^2^ for the ST100). For both filters, a sieving coefficient of ∼1.0 or more shows that meropenem is extensively filtered during CVVH. Vaborbactam filtration was also efficient, although the sieving coefficients were consistently lower than those of meropenem for all filters and conditions tested (mostly about 0.8 [Tables 2 and 3]). The relatively smaller molecular size of vaborbactam and no appreciable filter adsorption (Fig. 2) would seem to favor higher sieving coefficients than for meropenem; however, the difference in serum protein binding is perhaps the main factor for the observed lower vaborbactam sieving coefficient given that only free drug is available for filtration. Vaborbactam serum protein binding is about 33% (20), compared to only 2% for meropenem (22), which means that 30% less vaborbactam molecules are available for filtration. This is consistent with the 25% to 30% lower vaborbactam sieving coefficients observed, on average, for the ST100 (Table 2) and ST150 filters (Table 3), respectively. These observations are in agreement with previous studies that have demonstrated that for low- to middle-molecular-weight drug molecules, increasing plasma protein binding can limit the extent of filtration (23). Other factors, such as blood flow rate and effluent flow rate, did not appear to influence the sieving coefficient for either meropenem or vaborbactam. Similarly, the point of dilution did not result in sieving coefficient differences, with estimated values for prefilter versus postfilter dilution falling within 5% of each other given identical operational settings and consistently for both meropenem and vaborbactam (Table 2).

On the other hand, effluent flow rate significantly influenced filter clearance for both meropenem and vaborbactam, consistent with previous findings for meropenem (16, 24, 25) and other antibiotics (21, 24, 26, 27). The effluent flow rate affects the transmembrane pressure gradient, the main driver of convective removal of drugs in CVVH. With the ST150 filter, for example, there is a linear increase in the transmembrane pressure from 20 to ∼150 mm Hg with an increase in effluent rate from 0 to ∼4,000 ml/h (with postfilter dilution at a blood flow rate of 200 ml/min) (28). At higher effluent flow rates, the point of blood dilution has a further marked effect on the extent of clearance (Fig. 3). Prefilter fluid replacement results in lower clearance of both meropenem and vaborbactam because of the significant dilution of the blood, which therefore reduces the amount of drug passing through the filter per unit volume. The effect of such dilution is minimal at low effluent flow rates (hence replacement fluid flow rate); indeed, as illustrated in Fig. 3, filter clearances were comparable for pre- and postfilter dilution at the lowest flow rate tested (1,000 ml/h) for both meropenem and vaborbactam. However, a previous *ex vivo* study (14) did identify a statistically significant, but clinically insignificant, small increase in filter clearance with postfilter dilution (19.0 versus 17.6 ml/min) at an effluent flow rate of 1,000 ml/h. Similar to the effect of effluent flow rate, the relatively higher clearance at a higher blood flow rate (Fig. 4) is likely due to increased “fluid drag” during convective filtration in CVVH.

The lower rate of vaborbactam filter clearance seen in this study is perhaps an advantage for the meropenem-vaborbactam combination regimen to ensure adequate beta-lactamase inhibition for the entire course of the dosing interval, particularly in the treatment of infection caused by KPC-producing Enterobacteriaceae. A faster vaborbactam clearance is undesirable, as it would expose more molecules of meropenem to potential enzymatic degradation. Given the comparable *in vivo* pharmacokinetics of vaborbactam to that of meropenem (20), it would be reasonable to expect similar clearances by residual renal function during the clinical CVVH procedure, and thus, total vaborbactam clearance is unlikely to be greater than that of meropenem, maintaining an adequate relative ratio of the combination. However, a relatively more pronounced decrease in vaborbactam clearance during renal impairment (29) means that with the relatively lower filter clearance seen in this study, if dosing is optimized based on meropenem CRRT clearance only, vaborbactam accumulation may occur. Indeed, this could occur only after repeated dosing and during prolonged treatment courses, even during CVVH; however, available data suggest limited or no severe toxicities. We therefore suggest that future clinical studies should look into the relative concentrations of meropenem versus vaborbactam in patients receiving CVVH to describe any implications for safety. Of note, with hemodialysis, a recent study (29) showed a relatively higher increase in total clearance for vaborbactam (∼5-fold) versus meropenem (∼2-fold), suggesting that the different modalities may have different relative performance for vaborbactam versus meropenem clearance.

This *ex vivo* study is not without limitation. In the adsorption study, negligible mixing of blood with priming fluid during drainage from dead space was noted (a red tinge in the last 50 ml of drainage); this is unlikely to result in significant drug loss. In the clearance studies, large volumes of replacement fluid reduced mixing-chamber temperature. In addition, the amount of drug used in the *ex vivo* experiment is low compared to clinical dose, which resulted in a fast drop of mixing-chamber concentrations during sequential cycles of clearance experiments. The impact of this is that the initial nominal concentrations of 50 and 5 mg/liter were not maintained during subsequent cycles of the clearance experiments. Despite this limitation, pharmacokinetic estimates from the *ex vivo* study are predictive of filter clearance in patients undergoing CVVH (30) and provide data on a range of operational settings that would otherwise be difficult to generate in a clinical study.

In conclusion, no loss of vaborbactam dose occurs due to adsorption to commonly used CVVH circuit tubing and the AN69 ST100 filter, compared with minimal loss of meropenem. Both meropenem and vaborbactam are efficiently filtered with the AN69 ST100 or ST150 filter in the CVVH mode. Sieving coefficients and filter clearance for vaborbactam consistently are lower than for meropenem across different effluent flow rates and blood flow rates. The effluent flow rate appears to be the most important parameter affecting meropenem-vaborbactam filter clearance during CVVH. The effect of point of dilution on meropenem-vaborbactam filter clearance appears to be dependent on effluent rate: postfilter dilution is associated with increased filter clearance at higher effluent flow rates while having limited impact at low effluent flow rates. For standard durations of therapy, dosing according to existing meropenem data in CVVH is likely to be sufficient, i.e., 0.5 and 0.5 g or 1 and 1 g of meropenem and vaborbactam every 8 h for low effluent flow rates (1 to 2 liters/h) and high effluent flow rates (3 to 4 liters/h), respectively.

## MATERIALS AND METHODS

### Phase I: adsorption study. (i) Circuit setup.

A previously described CRRT circuit setup (31) was adapted for the adsorption experiment. This included a mixing chamber placed on a magnetic stirrer within an incubator (at 37°C). The mixing chamber was fitted with luer lock mixing cannulae as ports for connecting the access and return lines of a Prismaflex CRRT system (Gambro Lundia, Lund, Sweden) fitted with a ST100 set incorporating an AN69 ST hollow fiber acrylonitrile and sodium methallyl sulfonate copolymer filter (Gambro Industries, Meyzieu, France). The effluent outlet line was connected to a mixing cannula port on the mixing chamber, instead of drainage into an effluent bag, to set a recirculating closed circuit system. Saline was separately pumped into the effluent bag to prevent the Prismaflex aborting due to “a patient blood loss/gain” alarm.

### (ii) Preparation of blood-crystalloid mixture.

One unit of freshly collected (within 1 to 7 days) whole blood (approximately 500 ml) was combined with 1 ml of 5,000-IU heparin and sufficient Hartmann's solution (Baxter Healthcare, Toongabbie, Australia) to make a total volume of 1,000 ml. Human blood was sourced fresh and unfrozen from The Australia Red Cross Blood Service (Kelvin Grove, Australia) and kept chilled until use. The University of Queensland Human Research Ethics Committee B granted ethical clearance for the use of human blood (approval number 2017000460).

### (iii) Preparation of drug stock solutions.

Vials of meropenem-vaborbactam pharmaceutical preparation (containing 1 g of meropenem and 1 g of vaborbactam) were dissolved in sufficient distilled water (20, 50, or 200 ml) to obtain drug stock solutions of 50 mg/ml, 20 mg/ml, or 5 mg/ml. To prepare an antibiotic-containing blood-crystalloid mixture of a 50-mg/liter, 20-mg/liter, or 5-mg/liter concentration, from 1 liter of blank blood-crystalloid mixture, 1 ml was taken off prior to adding 1 ml of the 50-mg/ml, 20-mg/ml, or 5-mg/ml stock solution, respectively.

### (iv) Circuit priming and adsorption experiment.

Initially, the circuit was primed in slow continuous ultrafiltration (SCUF) mode (blood flow, 200 ml/min; simulated patient fluid removal, 2,000 ml/h). The priming fluid was a normal saline solution containing 5,000 IU/liter of heparin (citrate anticoagulation was not used). The mixing chamber was then filled with 1 liter of blood-crystalloid mixture (∼1:1 ratio of blood to crystalloid solution) containing meropenem and vaborbactam at concentrations of 50 and 50 mg/liter and 5 and 5 mg/liter. For each concentration, the adsorption study was conducted at blood flow rates of 200 ml/min and 100 ml/min. A blood sample was drawn immediately prior to the blood-crystalloid mixture being pumped into the circuit. When the blood-crystalloid mixture was first pumped, priming fluid equivalent to the internal volume of the circuit dead space was run to waste from the effluent end to prevent dilution, and the blood-crystalloid mixture was recirculated for 3 h. Serial blood samples (1 ml) were collected from the mixing chamber at 30, 60, 120, and 180 min for analysis of meropenem and vaborbactam concentrations. The adsorption experiment at each concentration (50/50 mg/liter and 5/5 mg/liter) was performed in three replicates.

In addition, a control stability experiment was performed in three replicates to quantify the amount of drug lost due to spontaneous degradation. In brief, blood-crystalloid mixture prepared at meropenem and vaborbactam concentrations of 50 and 50 mg/liter was kept in the mixing chamber at 37°C with constant stirring. Blood-crystalloid mixture samples for drug concentration measurement were collected at times identical to those in the adsorption experiments.

The percent drug lost by degradation was estimated from the stability experiment by using the following equation: percent drug lost by degradation = [1 − (measured concentration/initial concentration)] ×100.

The percent drug remaining after loss by adsorption and degradation was estimated from initial and measured concentrations during the adsorption experiment by using the following equation: percent drug remaining = (measured concentration/initial concentration) ×100.

The percent drug adsorbed at each time point was calculated by the following equation using mean percent drug lost by degradation and mean percent drug remaining: percent adsorbed = 100 − percent drug remaining − percent drug lost by degradation.

### Phase II: extracorporeal clearance study.

An *ex vivo* experimental model of CVVH was set up with a clinically used Prismaflex CRRT system (Gambro Lundia, Lund, Sweden). Unlike in the adsorption experiment, the ultrafiltrate was directed into the effluent bag and replacement fluid was introduced into the circuit by either predilution or postdilution. Priming fluid and blood-crystalloid mixture were prepared as described above. The Prismaflex with an AN69 filter set was primed in CVVH mode with the following settings: blood flow, 200 ml/min; pre-blood pump, 0 ml/min (no citrate anticoagulation); dialysis fluid, 0 ml/min (no dialysis); replacement fluid, 1,000 ml/min; percent predilution, 100%; and patient fluid removal, 0 ml/h (hence effluent flow rate matches the replacement fluid rate). Drug-spiked blood-crystalloid mixture was subsequently introduced with priming solution directed to waste, as described above, and the circuit was allowed to equilibrate for 20 min. Clearance experiments were run for different filters and settings in three replicates. Using an AN69 ST100 filter (nominal effective surface area of 1.0 m^2^) and initial meropenem and vaborbactam concentrations of either 50 and 50, 20 and 20, or 5 and 5 mg/liter, clearance experiments were run with a 2 × 3 CVVH setting matrix, including blood flow rates of 200 and 100 ml/min against replacement fluid (effluent) flow rates of 1,000, 2,000, and 4,000 ml/h. Similarly, using an AN69 ST150 filter (nominal effective surface area of 1.5 m^2^) and meropenem and vaborbactam concentrations of 50 and 50 mg/liter, clearance was assessed for a 1 × 3 CVVH setting matrix of blood flow rate (200 ml/min) against replacement fluid (effluent) flow rates (1,000, 2,000, and 4,000 ml/h). These experimental conditions were tested with both prefilter and postfilter introduction of replacement fluid.

For each specific setting, the circuit was allowed to equilibrate for 10 min prior to sampling. Samples were taken from the mixing chamber (1 ml of blood), the prefilter sampling port (1 ml of blood), the postfilter sampling port (1 ml of blood), and the effluent sampling port (1 ml of effluent). Blood samples were centrifuged (5 min at 15,000 rpm) and the plasma was stored frozen (−80°C) within 15 min. Effluent samples were transferred directly to labeled cryovials and frozen (−80°C) within 15 min. Samples were kept frozen until drug assay.

Extracorporeal clearance was calculated as follows: extracorporeal clearance = (effluent drug concentration/mixing chamber drug concentration) × effluent flow rate. The sieving coefficient was calculated as follows: sieving coefficient = effluent drug concentration/[(prefilter plasma concentration + postfilter plasma concentration)/2].

### Drug assay.

Total concentrations of meropenem and vaborbactam in plasma and renal replacement fluid effluent (RRTE) were measured by a validated high-performance liquid chromatography (HPLC)-tandem mass spectrometry (MS/MS) method on a Shimadzu Nexera2 ultrahigh-performance liquid chromatography (UHPLC) system coupled to a Shimadzu 8030+ triple quadrupole mass spectrometer. Each sample (plasma [10 μl] or RRTE [10 μl]) was spiked with internal standard (sulbactam and d6-meropenem) and proteins were precipitated with acetonitrile. An aliquot of 0.2 μl of the supernatant was injected onto the HPLC-MS/MS. The stationary phase was a Poroshell HPH-C18 2.1- by 50-mm, 2.7-μm column (Agilent, USA) operated at room temperature. The mobile phase A was 0.1% (vol/vol) formic acid in water, and mobile phase B was 100% acetonitrile with 0.1% (vol/vol) formic acid. The mobile phase was delivered with gradient from 5% to 50% at a flow rate of 0.4 ml/min for a run time of 5.5 min. Meropenem was monitored by positive mode electrospray at multiple reaction monitoring (MRMs) of 384.0 → 68.10 (384.0 → 141.10 as reference ion). Deuterated meropenem (d6-meropenem) was monitored in positive mode at 390.15 → 67.90 (390.15 → 147.00 as reference ion). Vaborbactam and sulbactam were monitored by negative mode electrospray at MRMs of 296.25 → 234.2 (296.25 → 278.15 as reference ion) and 232.35 →139.90, respectively. The method was validated for the calibration range from 0.05 to 100 mg/liter. Precision and accuracy of meropenem and vaborbactam were within the industry-standard limits of 15% at all four QC concentrations monitored (32).
